# The endo-lysosomal system of bEnd.3 and hCMEC/D3 brain endothelial cells

**DOI:** 10.1186/s12987-019-0134-9

**Published:** 2019-05-30

**Authors:** Andrea E. Toth, Simone S. E. Nielsen, Weronika Tomaka, N. Joan Abbott, Morten S. Nielsen

**Affiliations:** 1 Department of Biomedicine, Faculty of Health, Aarhus University, Ole Worms Allé 3, 8000 Aarhus, Denmark; 2 Lundbeck Foundation, Research Initiative on Brain Barriers and Drug Delivery, Scherfigsvej 7, 2100 Copenhagen, Denmark; 3 Institute of Pharmaceutical Science, King’s College London, 150 Stamford Street, SE1 9NH London, UK

**Keywords:** bEnd.3 mouse brain endothelial cell line, hCMEC/D3 human brain endothelial cell line, Primary porcine brain endothelial cell, Blood–brain barrier, Endo-lysosomal system, Vesicular transport, Intracellular trafficking, Lysosomes

## Abstract

**Background:**

Brain endothelial cell-based in vitro models are among the most versatile tools in blood–brain barrier research for testing drug penetration to the central nervous system. Transcytosis of large pharmaceuticals across the brain capillary endothelium involves the complex endo-lysosomal system. This system consists of several types of vesicle, such as early, late and recycling endosomes, retromer-positive structures, and lysosomes. Since the endo-lysosomal system in endothelial cell lines of in vitro blood–brain barrier models has not been investigated in detail, our aim was to characterize this system in different models.

**Methods:**

For the investigation, we have chosen two widely-used models for in vitro drug transport studies: the bEnd.3 mouse and the hCMEC/D3 human brain endothelial cell line. We compared the structures and attributes of their endo-lysosomal system to that of primary porcine brain endothelial cells.

**Results:**

We detected significant differences in the vesicular network regarding number, morphology, subcellular distribution and lysosomal activity. The retromer-positive vesicles of the primary cells were distinct in many ways from those of the cell lines. However, the cell lines showed higher lysosomal degradation activity than the primary cells. Additionally, the hCMEC/D3 possessed a strikingly unique ratio of recycling endosomes to late endosomes.

**Conclusions:**

Taken together our data identify differences in the trafficking network of brain endothelial cells, essentially mapping the endo-lysosomal system of in vitro blood–brain barrier models. This knowledge is valuable for planning the optimal route across the blood–brain barrier and advancing drug delivery to the brain.

**Electronic supplementary material:**

The online version of this article (10.1186/s12987-019-0134-9) contains supplementary material, which is available to authorized users.

## Background

The greatest obstacle to delivering drugs into the brain parenchyma is the presence of the blood–brain barrier (BBB), which limits molecular traffic between the blood and the nervous system. The morphological basis of the BBB is the monolayer of brain endothelial cells (BEC) in brain microvessels. The BECs are coupled tightly by intercellular junctions that significantly reduce permeation of ions and large hydrophilic solutes through the intercellular cleft via the paracellular pathway. Consequently, essential molecular delivery has to use vesicular pathways [1]. Receptor- and adsorptive-mediated transcytosis are responsible for the regulated vesicular transport of certain larger molecules including peptides, proteins and large pharmaceutical drugs [2, 3].

**Fig. 1:**
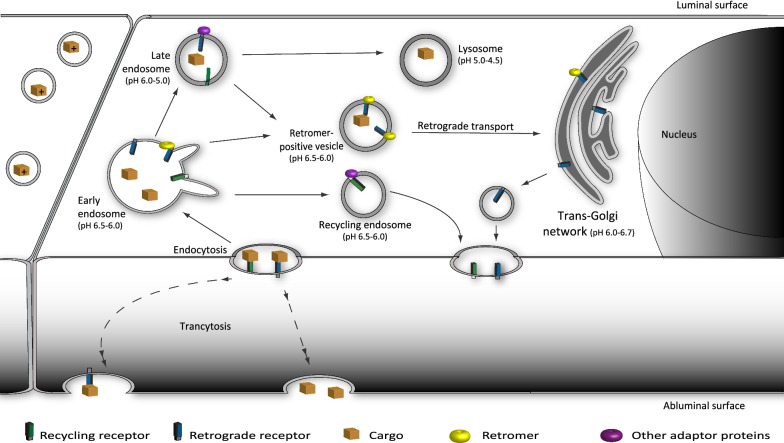
Vesicular transport in brain endothelial cells. The endogenous receptor-mediated transcytosis employs vesicular trafficking to transport ligands across the endothelium of the blood–brain barrier. This process involves the complex endo-lysosomal system. The endo-lysosomal system consists of trans-Golgi network, several types of vesicles such as early, recycling and late endosomes, retromer-positive vesicles and lysosomes. Early endosomes are the main sorting stations in the endocytic pathway, receiving receptors and cargos from almost all types of endocytosis. During vesicular sorting, internalized proteins, lipids and receptor–ligand complexes have three main destinations: (i) recycling back to the surface in recycling endosomes, (ii) retrograde trafficking to the trans-Golgi network in retromer-positive vesicles, or (iii) degradation in the lysosomes delivered by late endosomes. To facilitate receptor transport, the cell applies different types of cytosolic adaptor proteins e.g. adaptins or the retromers

Vesicular transport and transcytosis involves the complex endo-lysosomal system (Fig. 1) [4]. This system consists of the trans-Golgi network, several types of endosomal vesicle such as early, recycling, late endosomes and retromer-positive vesicles as well as lysosomes. Early endosomes are the main sorting stations in the endocytic pathway, receiving receptors and cargos from almost all types of endocytosis [5]. Besides cargos and receptors, early endosomes receive a large fraction of extracellular fluid and membrane components. These additionally internalized fluid and membrane, together with recycling receptors, are recycled back to the cell surface via recycling endosomes [6]. Meanwhile, certain ligands and retrograde receptors are retrograde transported toward the trans-Golgi network via retromer-positive vesicles. For the formation and sorting of retromer-positive structures, a multiprotein complex, the ‘retromer’ is responsible. The retromer complex is composed of a conservative cargo-recognition domain consisting of the vacuolar protein sorting-associated proteins (VPS), forming the VPS26–VPS29–VPS35 trimer and an additional variable domain of a sorting nexin dimer pair [7]. In polarized epithelial cells, the retromer also plays an important role in the transcytosis process [8]. Simultaneously, the remaining portion of early endosomes enters into the process of endosomal maturation and matures into late endosomes and finally into lysosomes [9]. Lysosomes are key organelles in degradation of a variety of biomacromolecules. The degradative function of lysosomes is carried out by over 60 luminal hydrolases with specificity for different substrates. Since the pH optimum of these hydrolases is highly acidic, lysosomes have the unique feature of containing the most acidic microenvironment (pH 4.5–5.0) inside the cells. The limiting membrane of lysosomes comprises more than 200 integral membrane proteins, including a proton-importing V-type ATPase that maintains the acidic pH of the lumen, and a set of highly-glycosylated lysosome-associated membrane proteins (LAMPs) that protect the membrane from degradation by the lysosomal hydrolases [10]. Lysosomal degradation represents a great challenge for drug targeting to the brain, since the majority of pharmaceutical candidates end up in lysosomes instead of being transcytosed across the BECs [3, 11].

Several studies have focused on potential drug delivery strategies of large pharmaceuticals and nanoparticles across the BBB using brain endothelial cell lines (for review see [1, 12]). Among these cell lines, the mouse bEnd.3 [13] and the human hCMEC/D3 [14] are two of the best-characterised and the most widely used for in vitro drug transport studies. The receptor expression and paracellular tightness of these cell lines are well described in the literature [15, 16]; however, their vesicular transport structures have been poorly investigated. Assumptions on their subcellular trafficking systems are based on observations from epithelial cell lines [3]. A recent study has pointed out that subcellular trafficking in primary BECs differs in detail from that of epithelial cells and therefore needs to be specifically examined [17]. Therefore, our aim was to investigate and characterize the endo-lysosomal system in the mouse bEnd.3 and the human hCMEC/D3 cell lines, which are widely used for the investigation of receptor-mediated transcytosis in in vitro drug transport studies. In our previous study, we already described and characterized the endo-lysosomal structure of primary porcine BECs (PBEC) [17]. Consequently, we compare our observations on the cell lines to the PBEC model.

## Methods

### Reagents

All reagents and chemicals were purchased from Sigma-Aldrich (Rødovre, Denmark) unless otherwise indicated.

### Cell cultures

The investigated BEC cell lines were cultured according to the manufacturer’s recommendations as used by most laboratories. The culture medium used has been optimised for each BEC model to strengthen and maintain their BBB attributes [13, 18, 19].

The mouse brain endothelial cell line, bEnd.3 (ATCC^®^ CRL2299™; Manassas, VA, USA) was used between passage 22 and 29. The cells were cultured in Dulbecco’s Modified Eagle Medium (DMEM) supplemented with 10% fetal bovine serum (FBS) and 5 μg/ml gentamycin [13]. The medium was refreshed on every 3rd day. Cells were divided and seeded at a density of 5 × 10^4^ cells/cm^2^ on collagen IV (500 µg/ml) and fibronectin (100 µg/ml) coated cover glasses, 8-well chamber µ-slides (Ibidi, Ramcon A/S, Birkerød, Denmark) or in 4-well cell culture dishes (Nucleon Delta Surface Thermo Fisher Scientific, Roskilde, Denmark). The cells grow to confluent monolayers within 3 days after seeding.

The human brain endothelial cell line, hCMEC/D3 (Millipore Sigma, Denmark) was used in the experiments between passage number 30 and 35. Cells were cultured in EBM-2 medium (Lonza, Walkersville, MD, USA) containing 5% FBS, hydrocortisone (1.4 mM), 10 mM HEPES ((4-(2-hydroxyethyl)-1-piperazineethanesulfonic acid) pH 7.4, gentamycin (50 mg/ml), ascorbic acid (5 mg/ml), 1% chemically-defined lipid concentrate, basic fibroblast growth factor (1 ng/ml) on rat tail collagen type I (30 µg/ml)-coated T75 flasks. The medium was changed every 3rd day. Depending on the experimental setup, the cells were seeded on coated cover glasses, 8-well chamber slides or in 4-well cell culture dishes at a density of 2.5 × 10^4^ cells/cm^2^. When cells reached approximately 70–80% confluency the medium was supplemented with 10 mM lithium chloride [19]. Cells became completely confluent within 4 days.

Isolation of porcine brain microvessels was carried out as described in detail in an earlier published protocol from our laboratory [18]. Following isolation, porcine brain capillaries were plated in T75 flasks coated with collagen IV (500 µg/ml) and fibronectin (100 µg/ml). Cells were cultured in DMEM/F12 medium supplemented with 10% plasma-derived bovine serum (PDS; First Link Ltd, Wolverhampton, UK), basic fibroblast growth factor (1 ng/ml), heparin (15U), insulin-transferrin-selenium (100 µg/ml) and gentamicin (5 μg/ml). Puromycin (4 μg/ml) was added to the medium for the first 3 days in order to obtain a pure culture of PBEC and remove contaminating cells. Cells were grown until 70% confluency then passaged onto coated cover glasses, 8-well chamber slides or in 4-well cell culture dishes for experiments. Cells were seeded at a density of 1–2 × 10^5^ cells/cm^2^. When the PBEC had reached confluence—approximately 2 days after seeding—the medium was supplemented with differentiation factors; 550 nM hydrocortisone, 250 μM 8-(4-chlorophenylthio)adenosine-3′,5′-cyclic monophosphate (cAMP) and 17.5 μM RO-201724. All cell cultures were maintained in a humidified incubator with 5% CO_2_ at 37 °C.

### Antibodies

All antibodies used in this study are commercially available and listed in Additional file 1. Specificity of the antibodies was confirmed for all three cell types by Western blot (Additional file 2). These antibodies are commercially available and have previously been used as specific markers for investigation of trafficking and vesicular structures in our own [17] and several other laboratories. Additionally, the cell borders of BEC were marked with antibody against adherens junction protein p120 catenin.

### Western blot

For cell lysate, BEC were cultured in coated T75 flask as described above. The confluent cell layers were washed with phosphate-buffered saline (PBS) and lysed in ExB lysis buffer (150 mM NaCl, 20 mM MgCl_2_, 20 mM CaCl_2_, 100 mM HEPES, 1% TritonX-100, complete protease inhibitor). 2.7 μg of each protein sample was loaded on a 4–12% polyacrylamide gel (Novex NuPage, Thermo Fisher Scientific) and subsequently transferred to nitrocellulose membrane. Membranes were blocked in 5% skimmed milk, 0.01 M Tris–HCl, 0.15 M NaCl, and 0.1% Tween 20, pH 7.6 in buffer solution at room temperature (RT). Primary antibodies (1:1000) were applied overnight at 4 °C. On the following day, membranes were incubated with HRP-conjugated secondary antibodies (1:2000) for 1 h at RT. Specific band size was detected with ECL (GE Healthcare, Brøndby, Denmark) or SuperSignal (Thermo scientific, Rockford, USA) according to the manufacturer’s recommendations and visualised using LAS 4000 (Fujifilm).

### Immunocytochemistry and confocal microscopy

Cells for immunocytochemistry were grown on coated cover glasses as described above. Confluent cell layers were fixed with 4% paraformaldehyde in PBS at RT, or for RAB7 staining with methanol for 10 min at − 20 °C. The further steps were carried out at RT. For permeabilisation and blocking of the samples, we used 0.3% Triton-X100 and 1% bovine serum albumin in PBS for 20 min. Primary antibodies were diluted in 1:300 and secondary antibodies in 1:400 of the above-mentioned solution. With both primary and secondary antibodies, samples were subsequently incubated for 1 h. Alexa-Fluor-488 conjugated secondary antibodies were used against the primary antibodies of the vesicular structures. For staining of the nuclei, Hoechst 32528 (0.5 μg/ml) in distilled water for 10 min was applied as a separate step. In between the steps, samples were washed 3 times for 5 min in PBS to remove unbound antibodies. Finally, samples were mounted on glass slides using Dako fluorescence mounting medium (Dako, Glostrup, Denmark). Further steps were carried out by high content microscopy as described below.

Representative confocal images were captured by Olympus IX-83 fluorescent microscope with Andor confocal spinning unit and Andor iXon Ultra 897 camera, Olympus Upsalo W, 60×/1.20 NA water objective lens, using Olympus CellSens software (Olympus). Multichannel images were processed using Fiji software. Brightness and contrast adjustments were applied for the channel independently.

### Lysosomal acidification

The intralysosomal acidification was estimated using LysoSensor Green DND-189 dye (Invitrogen, Thermo Fisher Scientific). The LysoSensor dyes are acidotropic probes that accumulate in acidic organelles as in matured late endosomes and lysosomes. The dyes’ fluorescence intensity shows inverse correlation with the pH-value [20]. Since LysoSensor Green DND-189 has a low pKa value (5.2), it is nonfluorescent except when inside acidic compartments. To verify the sensitivity of the LysoSensor probe, we incubated the cells with or without 100 nM bafilomycin A1 for 45 min before LysoSensor uptake and during the measurement (Additional file 3). For the experiments, cells were grown in 8-well chamber slides with the above-mentioned culture conditions. When BEC reached the desired confluence, the cells were incubated with prewarmed media containing 1 µM LysoSensor and 0.125 μg/ml Hoechst 32528 for 15 min at 37 °C. Then the cells were washed twice with PBS and kept in FluoroBrite DMEM media (Gibco, Thermo Fisher Scientific) for imaging. Samples were immediately observed in a microscope equipped with a live cell imaging chamber in humidified atmosphere with 5% CO_2_ at 37 °C. Fluorescent intensity was measured with a fluorescence confocal microscope (Olympus BX73 microscope) fitted with the correct filter set as described below. We measured the relative mean fluorescence intensity in at least 30 images per sample.

### High content screening analysis

Following immunocytochemistry and LysoSensor dye uptake, images for high content screening were obtained with the Olympus automated Scan^R high content imaging station based on an Olympus BX73 microscope, with a 60×/0.9 NA air objective, triple-band emission filter for Hoechst 33258, Alexa-Fluor-488 and Alexa-Fluor-568, and a Hamamatsu camera (C8484-05G). Image analysis was performed using Scan^R image and data analysis software for Life Science (Münster, Germany) as described previously [17, 21]. Briefly, single-layer images were background-corrected and edge-detection algorithm was applied to segment subcellular structures based on detection of gradient intensities of the chosen colour channel. The software segmented subcellular structures independently if a closed connecting line (edge) could be drawn around them and their area was larger than 0.05 µm^2^ independently of their shape. Images with artefacts or out of focus were manually gated out. The total number of vesicles was normalized to the number of nuclei before making comparison among the adjacent groups. The distance between the objects was determined by applying Pythagoras’ theorem on x; y coordinate values of the objects’ border. Based on the lateral distance from the nucleus, subcellular zones were defined inside the cells (Additional file 4): The juxtanuclear zone covers the area of nuclei and 1 µm around it. The peripheral zone of the cells was delineated between 1 and 2 µm distance from the nucleus. The third zone was named projection, since this subcellular region encompasses the flat elongated projections (fine processes) of the cells. Number, area, morphology and fluorescent intensity of vesicles from 3500 to 5500 cells for each group were analysed.

### Lysosomal degradation activity measurement

The 40 kDa receptor-associated protein (RAP), a ligand for members of the low density lipoprotein receptor family [22], and to the heparan sulfate [23], was radioactively labeled with Ci ^125^I (^125^I-RAP) using a Sepharose G25 column [24]. The column material was packed in a 2 ml syringe with glass wool in the bottom and eluted with 1% bovine serum albumin (BSA) in PBS, pH 7.4. A solution of 50 µl 0.2 M NaH_2_PO_4_ pH 8.0, 5 µg RAP, 3 µl 2 M Ci ^125^I, 5 µl 0.5 mg/ml Chloramin T, and 5 µl 0.5 mg Na_2_S_2_O_5_ was added to the column. Eluent fractions were collected as three drops per tube and stored at − 20 °C. For the experiment, cells were grown in 4-well dishes for approximately 2–4 days until reaching confluency. Before the experiments, the cell culture medium was refreshed (700 μl/well) and later an additional 100 μl medium supplemented with Ci ^125^I-labelled RAP (approximately 30,000 counts per million (CPM)/100 μl) was added to the wells. Wells with or without cells were incubated for 1, 3, 6 and 24 h respectively. After incubation, the medium was collected, and NaOH was added to the cells to allow counting of cell-associated ^125^I-RAP. Following NaOH addition, cells were incubated for 10 min at RT, and the cell solution transferred to counting tubes. The intact ^125^I-RAP was precipitated by addition of 2.5 ml 12.5% trichloroacetic acid and 100 µl 10% BSA in distillated water and centrifuged at 3000*g*, 4 °C for 10 min. Degradation of ^125^I-RAP was assessed by measuring radioactivity in the resulting supernatant using a Packard Cobra Gamma 5002 counter reader (GMI, Ramsey, Minnesota, USA). The percentage of degraded ^125^I-RAP was calculated from the total CPM read after subtracting the cell-associated ^125^I-RAP, followed by adjustment for the cell number.

### Statistical analysis

All experiments were repeated at least three times in triplicate for each group. All data are presented as mean ± SEM. Values were compared using one-way ANOVA followed by Tukey’s multiple comparison *posthoc* tests using GraphPad Prism 7.0 software (GraphPad Software Inc., San Diego, CA, USA). Changes were considered statistically significant at *p* ≤ 0.05.

## Results

**Fig. 2:**
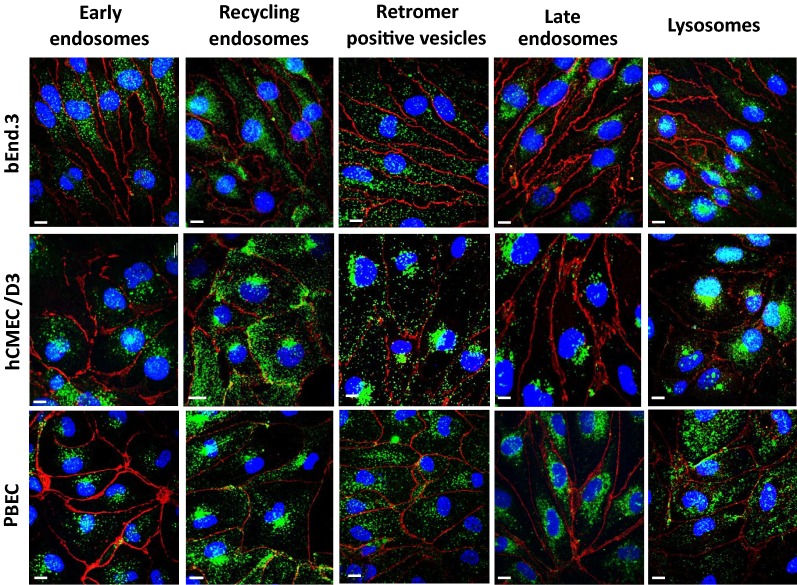
Representative confocal microscopy images of vesicular structures (green) in brain endothelial cells. The junctions of the cells were stained against p120 catenin (red). Nucleus is marked in blue. Magnification is 60×. Scale bar: 10 µm

To classify and quantify the different types of vesicle (Fig. 1), early endosome antigen 1 (EEA1) was targeted as a specific marker for early endosomes, transferrin receptor (TfR) for recycling endosomes, VPS35 for the retromer-positive vesicles, Ras-related protein 7 (RAB7) for late endosomes and LAMP1 for lysosomes (Fig. 2), as described previously [17]. During high-content screening analysis, the number of vesicles was normalized to the number of nuclei before further comparisons. The number (Fig. 2) and size of the nuclei (area; bEnd.3 149.80 ± 9.77 µm^2^, hCMEC/D3 170.08 ± 4.06 µm^2^, PBEC 140.48 ± 22.73 µm^2^, diameter; bEnd.3 13.71 ± 0.45 µm, hCMEC/D3 14.69 ± 0.17 µm, PBEC 12.74 ± 0.93 µm) on the images did not differ significantly among the investigated groups.

### Number of vesicles

**Fig. 3:**
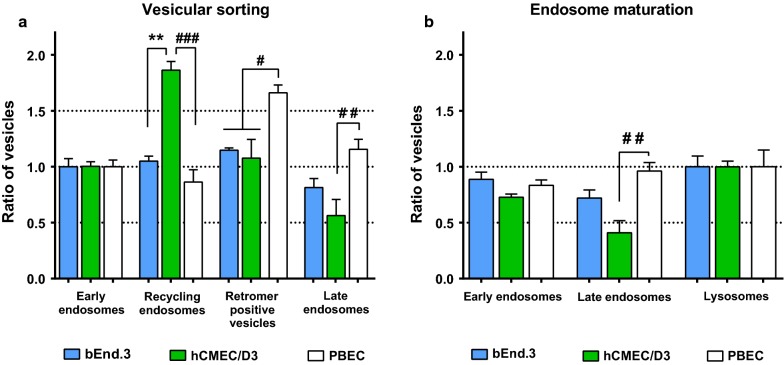
Ratio of vesicular structures. **a** The amount of vesicles per cell is shown normalised to the number of early endosomes. **b** The amount of endosomes per cell is presented relative to the number of lysosomes. All values are presented as mean ± SEM, n > 9. Statistical analysis; Difference was analysed by one-way ANOVA followed by Tukey’s posthoc test. Values were considered statistically significant at *p ≤ 0.05, **p ≤ 0.01 between the cell lines (bEnd.3 vs. hCMEC/D3) and at ^#^*p* ≤ 0.05, ^##^*p* ≤ 0.01, ^###^*p* ≤ 0.001 compared to the primary PBEC

Early endosomes function as sorting stations hence they are the starting point for vesicular recycling, retrograde transport and for endosome maturation [5]. Therefore, we normalised the number of recycling, retromer-positive vesicles and late endosomes to the number of early endosomes in each cell type (Fig. 3a). In the hCMEC/D3 cells the number of recycling endosomes was double the number of early endosomes, while the late endosomes were only half the number of the early endosomes. This ratio pattern differed significantly from those of the PBEC and from the bEnd.3 cells. By contrast, the primary PBEC possessed a significantly higher amount of retromer-positive structures than the cell lines.

The number of lysosomes was similar and did not differ significantly among the investigated groups (Table 1). Since lysosomes are the end point of endosome maturation [25], we normalized the number of the other participants of the process—i.e. early and late endosomes, to the number of lysosomes in each cell type. Generally, each cell type contained fewer early endosomes than lysosomes. By comparing the ratio of vesicles among the groups, we found that the human cell line had a lower amount of late endosomes than the other BECs (Fig. 3b).

**Table 1:** Number of vesicles per cell

	bEnd.3	hCMEC/D3	*p*	PBEC	*p* ^a^	*p* ^b^
	Mean ± SEM	Mean ± SEM		Mean ± SEM		
Early endosomes	215.98 ± 15.81	147.58 ± 6.18	*	155.71 ± 9.02	#	ns
Recycling endosomes	226.74 ± 9.94	274.80 ± 11.34	ns	134.02 ± 17.00	# #	# # #
Retromer-positive structures	247.18 ± 4.50	158.82 ± 24.74	*	258.64 ± 10.60	ns	# #
Late endosomes	175.83 ± 17.44	83.19 ± 21.45	*	179.77 ± 14.23	ns	# #
Lysosomes	242.88 ± 23.39	202.89 ± 10.31	ns	186.85 ± 27.89	ns	ns

Values are presented as mean ± SEM, n > 9. Statistical analysis: Difference was analysed by one-way ANOVA followed by Tukey’s *posthoc* test

Values were considered statistically significant at * p ≤ 0.05, ** p ≤ 0.01, *** p ≤ 0.001 between the cell lines (bEnd.3 vs. hCMEC/D3) # p ≤ 0.05, ## p ≤ 0.01, ### p ≤ 0.001

*ns* not significant, *p*^a^ significance when PBEC compared to bEnd.3, *p*^b^ significance when PBEC compared to hCMBEC/D3

When comparing the raw number of vesicles per cell between the cell lines (Table 1), the amount of early, late endosomes and retromer-positive vesicles was significantly lower in the hCMEC/D3 than in the bEnd.3. On the other hand, the number of recycling endosomes and lysosomes was similar and did not differ significantly. By contrast, the primary PBEC had markedly fewer recycling endosomes than any of the cell lines. The PBEC possessed more late endosomes and retromer-positive structures than the hCMEC/D3, similarly to bEnd.3. However, the number of early endosomes was significantly lower than in bEnd.3 and was similar to that of hCMEC/D3.

### Subcellular lateral distribution of the vesicles

**Fig. 4:**
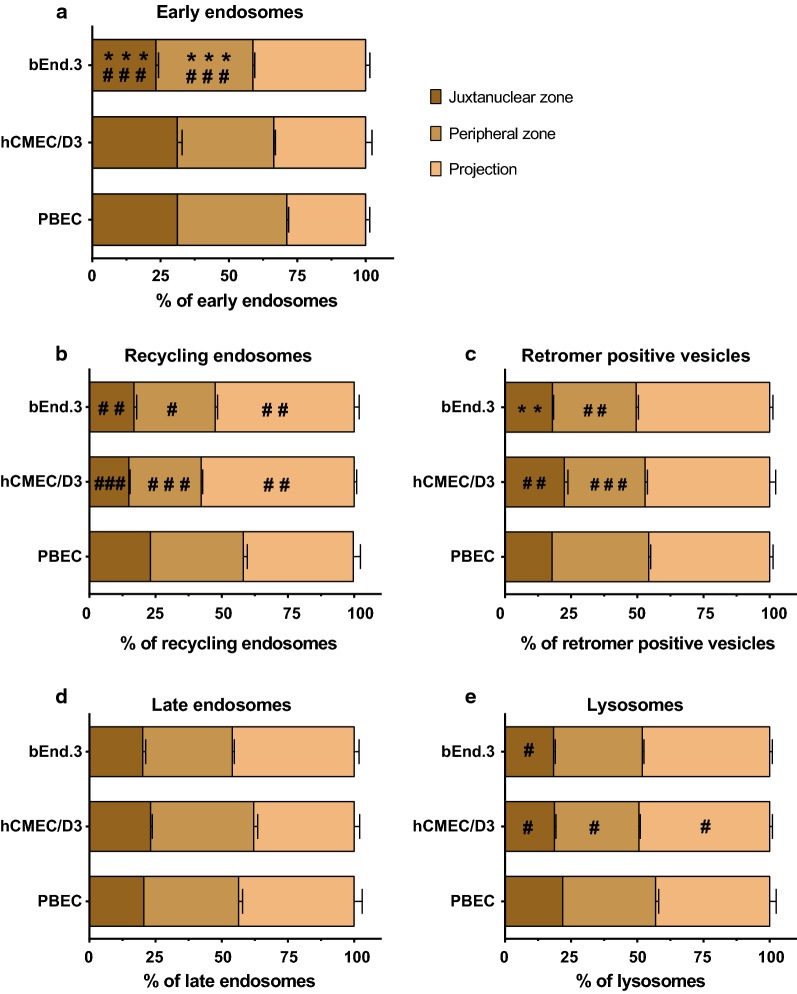
Subcellular lateral distribution of vesicles in the subcellular zones. The total number per cell for each vesicle type was considered as 100%. Values are presented as mean, n > 9. Statistical analysis; Difference was analysed by one-way ANOVA followed by Tukey’s *posthoc* test. Values were considered statistically significant at *p ≤ 0.05, **p ≤ 0.01, ***p ≤ 0.001 between the cell lines (bEnd.3 vs. hCMEC/D3) and at ^#^*p* ≤ 0.05, ^##^*p* ≤ 0.01, ^###^*p* ≤ 0.001 compared to the primary PBEC

We detected differences in the ratio or the number of all vesicle types, except for the lysosomes (Fig. 3 and Table 1). However, the location of the lysosomes, even without a difference in their number inside the cells, can indicate different physiological function [26]. Therefore, we investigated and compared the lateral distribution of lysosomes and other endosomes inside the BEC. Each cell was divided into subcellular zones (Additional file 4) and the proportion of the vesicles relative to their total number (100%) inside the cell calculated (Fig. 4). Early endosomes showed a different distribution compared to the other vesicles; they were close to evenly distributed among the three subcellular zones (Fig. 4a). Other than that, the lowest proportion of vesicles was found in the juxtanuclear zone. Less than 25% of recycling endosomes, retromer positive vesicles, late endosomes and lysosomes were located in this subcellular zone. The majority (~ 50%) of these vesicles occupied the projections of the cells—more than 3 µm away from the nucleus (Fig. 4b–e).

When comparing the pattern among the BEC, we observed several differences in distribution of the vesicles with the exception of late endosomes (Fig. 4). Interestingly, the spread of late endosomes did not differ significantly among the groups (Fig. 4d). The proportion of early endosomes was similar between the primary PBEC and the human hCMEC/D3 cell line and was markedly distinct from that of the bEnd.3 (Fig. 4a). In contrast, the spread of recycling endosomes, retromer-positive vesicles and lysosomes differed significantly in both cell lines from the pattern in primary PBEC and was similar in the two cell lines (Fig. 4b, c, e).

### Morphometric analysis; shape and area

**Fig. 5:**
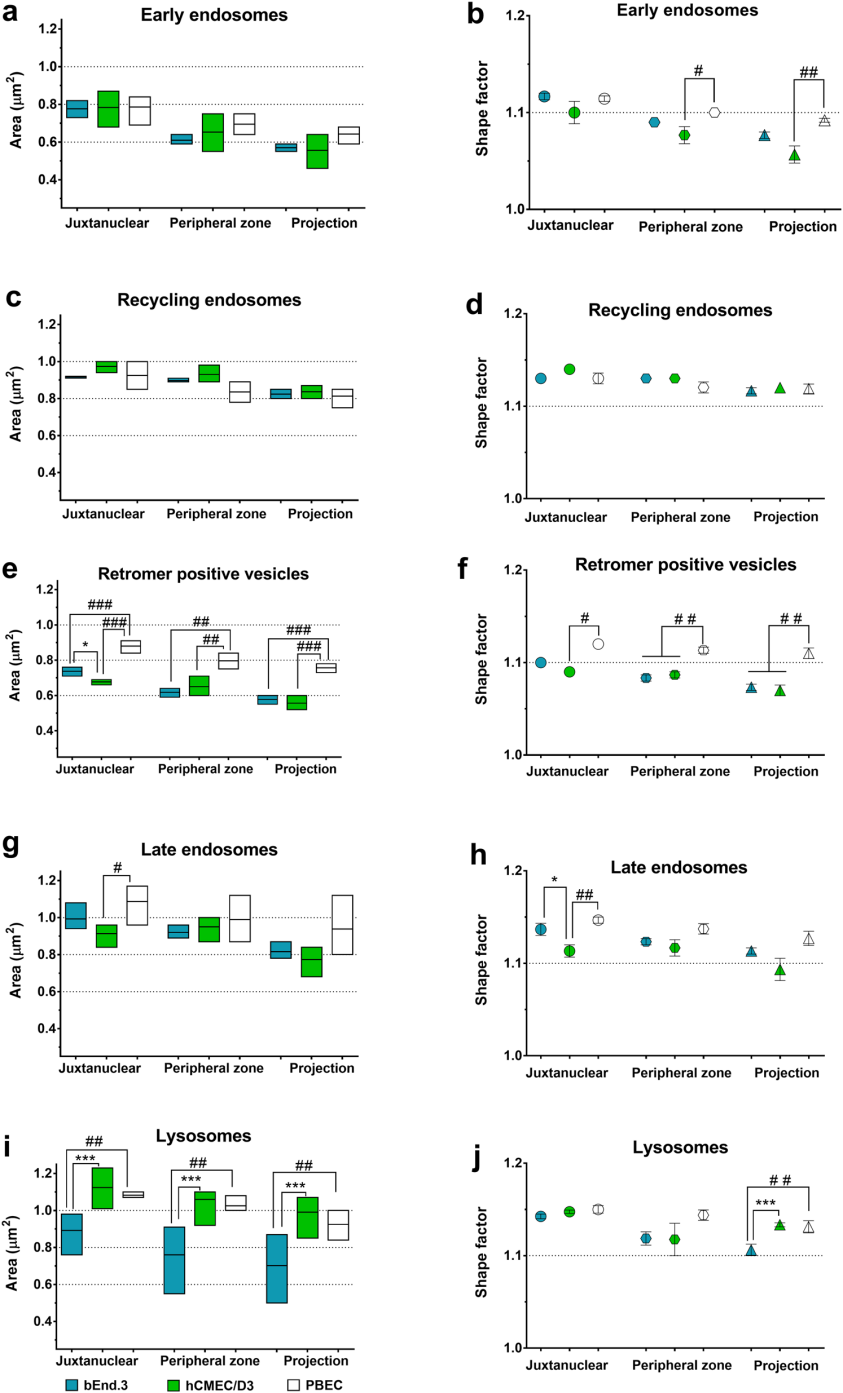
Morphometric analysis: area and shape. **a**, **c**, **e**, **g**, **i** The area of the vesicles is shown with a box diagram (left panel). The box represents 25 and 75 percentiles. The horizontal line represents the mean. **b**, **d**, **f**, **h**, **j** The shape factor (circularity) describes the shape of adjacent vesicles. Values are presented as mean ± SEM, n > 9. Statistical analysis; Difference was analysed by one-way ANOVA followed by Tukey’s *posthoc* test. All values were considered statistically significant at *p ≤ 0.05, **p ≤ 0.01, ***p ≤ 0.001 between the cell lines (bEnd.3 vs. hCMEC/D3) and at ^#^*p* ≤ 0.05, ^##^*p* ≤ 0.01, ^###^*p* ≤ 0.001 compared to the primary PBEC

Since not only the amount of vesicles, but also their size and shape can vary [4, 25], we investigated their area and their shape factor (circularity) in the different subcellular zones of the BECs (Fig. 5). Interestingly, a tendency to decreasing area could be observed in all vesicle types as a function of their distance from the nucleus (Fig. 5 left panel). The shape factor of the investigated vesicles varied between 1.05 and 1.15. These values define irregular shapes (Fig. 5 right panel), since the circularity factor of the perfect circular shape is 1.00.

When comparing the groups, the most remarkable differences were observed in retromer-positive vesicles and lysosomes (Fig. 5e, f, i, j). The retromer-positive vesicles in PBEC were larger than those in the cell lines and their shape factor was significantly different. These vesicles in the cell lines had the same size and similar shape (Fig. 5e, f). By contrast, the lysosomes of PBEC and hCMEC/D3 were larger than those in b.End3. However, lysosomes in b.End3 cells showed the greatest variation in size among all the vesicles (Fig. 5e). Furthermore, the circularity factor of lysosomes in the projections differed significantly from the b.End3 and was similar between the hCMEC/D3 and the PBEC (Fig. 5f).

### Lysosomal function

**Fig. 6:**
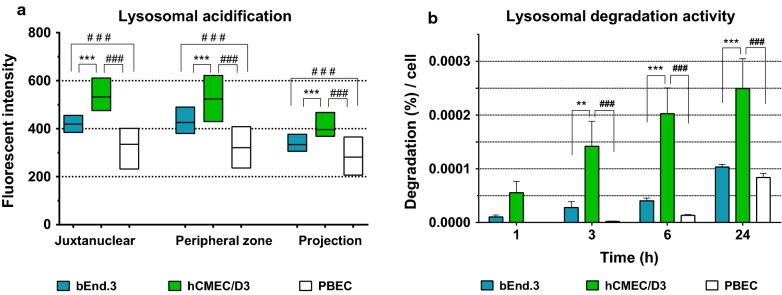
Lysosomal function. **a** Fluorescent intensity of Lysosensor green shows the acidification of lysosomes and matured late endosomes. Data are shown with box diagrams; the horizontal line represents the mean. The border of the box represents the error. **b** The bar graph shows the percentage of degraded ^125^I-RAP per cells at different time points. Values are presented as mean ± SEM, n > 9. Values were considered statistically significant at *p ≤ 0.05, **p ≤ 0.01, ***p ≤ 0.001 between the cell lines (bEnd.3 vs. hCMEC/D3) and at ^#^*p* ≤ 0.05, ^##^*p* ≤ 0.01, ^###^*p* ≤ 0.001 compared to the primary PBEC

**Fig. 7:**
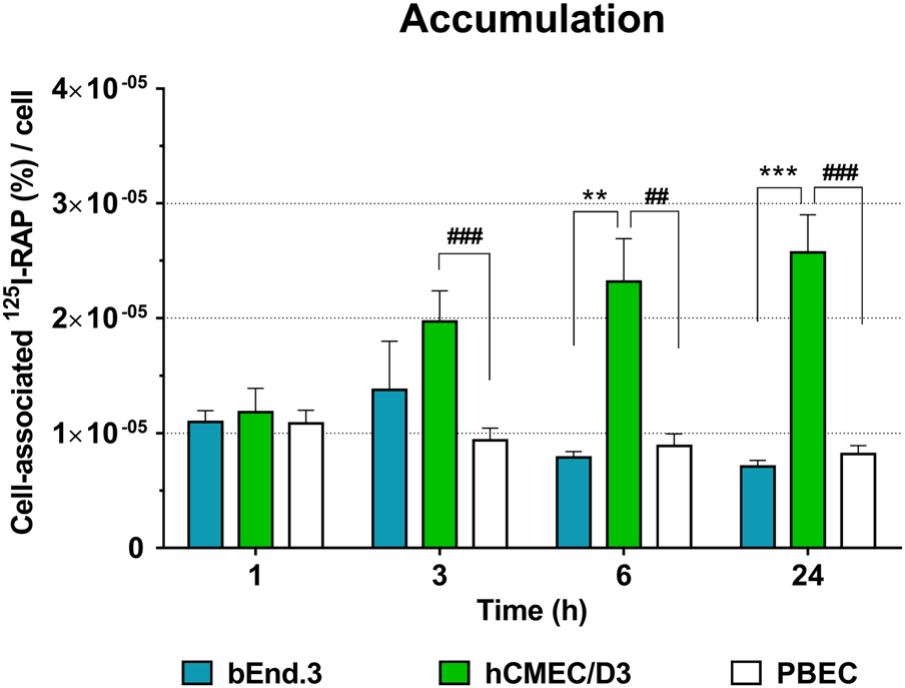
Accumulation. The bar graph shows the percentage of cell-associated ^125^I-RAP per cell at different time points. Values are presented as mean ± SEM, n > 9. Values were considered statistically significant at *p ≤ 0.05, **p ≤ 0.01, ***p ≤ 0.001 between the cell lines (bEnd.3 vs. hCMEC/D3) and at ^#^*p* ≤ 0.05, ^##^*p* ≤ 0.01, ^###^*p* ≤ 0.001 compared to the primary PBEC

To evaluate the function of lysosomes, we measured the acidification of late endosomes and lysosomes (Fig. 6a) and degradation of ^125^I-RAP over time (Fig. 6b). Lysosomes and matured late endosomes enclose a highly acidic environment within the cells (Fig. 1). We found that hCMEC/D3 possess the most acidic organelles in all subcellular zones of the cells compared to bEnd.3 and PBEC (Fig. 6a and Additional file 3). The matured late endosomes and lysosomes of bEnd.3 also showed higher fluorescent intensity than those of the PBEC, but the intensity was significantly lower than in the hCMEC/D3 in all parts of the cells. Generally, the less acidic vesicles were located in the projections of the cells and the most acidic ones with higher fluorescent intensity were closer to the nucleus in all groups of BEC. Treatment with bafilomycin A1, a specific V-ATPase pump inhibitor [27] was used to verify the exclusive fluorescent property of the dye for acidophilic components in all cell types (Additional file 3). We could not detect fluorescent organelles in any of the cells in the presence of bafilomycin, confirming that it abolished the operation of the proton pumps responsible for creation of low pH inside the vesicles. In accord with our observations on lysosomal acidification, we measured the highest lysosomal degradation activity of RAP in hCMEC/D3 over time (Fig. 6b). In the case of bEnd.3 cells, the amount of degraded ^125^I-RAP was high compared to PBEC, but lower than the degradation by hCMEC/D3. Within the 1st h, we could not detect differences in the amount of cell-associated ^125^I-RAP, indicating no difference in the binding affinity of the ligand among the BECs. However, at later time points, we could see significantly high accumulation of the ligand in hCMEC/D3 (Fig. 7). Altogether, we measured the lowest level of acidification and smallest amount of degraded ^125^I-RAP protein in the primary PBECs (Fig. 6).

## Discussion

Our study classified and quantified the intraendothelial vesicles and investigated certain aspects of the lysosomal function in different in vitro BEC models. A large number of BBB culture models are used in basic as well as applied research and detailed characterization and comparative datasets are needed to select the appropriate model for drug delivery studies. However, such studies are scarce. The present work on two cell line-based models in comparison with primary PBEC is unique; no such comparative study of the endo-lysosomal system of BEC has previously been published.

Our findings complement previous studies on receptor-mediated transcytosis and transcellular permeability in bEnd.3 [13, 28–30] and hCMEC/D3 cells [14, 31, 32]. These studies concluded that the cell line models of the BBB do not form as tight a barrier as the primary BEC cells. Generally, the TEER values of the cell lines are relatively low and permeability measured by paracellular markers are significantly high when compared to primary BEC systems [13, 31, 33]. On the other hand, they are suitable models for large scale drug transport studies of large molecules based on their receptor expression pattern, surface charge and transcellular properties [13–15, 31]. Here we have shown that the essential subcellular organelles of vesicular trafficking (Fig. 1) are present in all three types of BBB models (Fig. 2), although their ratio and attributes differ.

Early endosomes are the initial sorting stations after endocytosis, therefore they are localised mainly in the perimeter of the cells [34]. The bEnd.3 cell line possessed the highest amount of early endosomes (Table 1) and as expected had the highest portion of these endosomes in the projections compared to the other BEC (Fig. 4). Our finding is in agreement with a previous study on early endosomes in primary BEC, where the elevated number of endosomes was more dominant in the peripheral cytoplasm of the cells [17].

During vesicular sorting, internalized proteins, lipids and receptor–ligand complexes have three main destinations: recycling back to the surface, retrograde trafficking to the trans-Golgi network, or degradation in the lysosomes (Fig. 1). We observed that the ratio of vesicles designated to these destinations were markedly different in hCMEC/D3 cells (Fig. 3). The hCMEC/D3 has twice as many recycling endosomes and half the number of late endosomes of the other BECs when compared to the amount of early endosomes (Fig. 3a) or lysosomes (Fig. 3b). These results demonstrate that hCMEC/D3 cells have more cell organelles for the recycling pathway than for degradation of the cargo. This observation was confirmed by the accumulation of ^125^I-RAP ligand in these cells (Fig. 7). In particular, the recycling pathway seems to be preferred in hCMEC/D3. This is particularly interesting, since several laboratories target the recycling receptors of BEC such as TfR and low-density lipoprotein receptor-related protein 1 (LRP-1) for drug delivery (for review see [1–4]).

Interestingly, retromer-positive vesicles have markedly distinct attributes in the primary BEC compared with the cell lines; these vesicles were larger and their shape was more irregular in PBEC (Fig. 5). Additionally, the number of retromer-positive vesicles was significantly higher when normalized to the number of early endosomes than in the cell lines (Fig. 3a). Retrograde-transported receptors represent a new and exciting target for drug delivery to the brain, particularly since the retrograde-receptor mannose-6-phosphate receptor has been described in PBEC as a potential target for receptor-mediated transcytosis [35]. Our findings should be taken into consideration when choosing an appropriate in vitro model for investigation of retromer-transported ligands.

Lysosomes are one of the most interesting members of the endo-lysosomal system, since they play a central role in the control of cell metabolism (for review see [36]). They fulfil most of these functions via intracellular degradation, therefore, we focused here on their degradative function. We found that the lysosomes of hCMEC/D3 could break down the highest amount of radioactive-labelled ligands over time, followed by the other cell line, bEnd.3 (Fig. 6b). According to this result, the cell lines possessed more acidic organelles than the PBEC (Fig. 6a). This phenomenon could be explained by the ratio of lysosomes to late endosomes (Fig. 3b). Matured late endosomes are also acidic organelles of the cells (pH 5.5–5) but to a lesser extent than lysosomes (pH 5–4.5). PBEC contained equal numbers of lysosomes and late endosomes, however in the cell lines the late endosomes were greatly outnumbered by lysosomes (Fig. 3b). Interestingly, the number of lysosomes was equal among the investigated BECs (Table 1), but they were of larger size in PBEC and hCMEC/D3 than in bEnd.3 (Fig. 5i). The amount of lysosomes seems to be a constant factor in BECs (Table 1), since even astrocytes are not able to influence their number [17]. The reason behind this phenomenon could be the essential role of lysosomes in cell metabolism, but further investigation is needed to reveal the exact involvement of lysosomes in BEC functions. Knowledge of lysosomal activity is important to consider when studying transcytosis of low-affinity receptor ligands, since they may be released from their receptor(s) in the acidic environment of endosomes. One such example is the many low-affinity transferrin receptor antibodies uses by several groups attempting to deliver therapeutic antibodies to the brain.

Our study aimed to provide quantitative and statistical information on the endo-lysosomal composition of those BECs that are frequently used for investigation of drug transport as in vitro models of BBB. Most laboratories in academia as well as in industry choose the models that offer the best combination of convenience, cost and applicability to their research questions [12, 37]. Despite the fact that primary models are thought to represent more closely the in vivo circumstances, immortalized cell lines serve as simple and non-expensive tools for CNS drug delivery and discovery research [12]. However, the primary PBEC model is comparable to the cost efficiency of cell lines, because the abattoirs are a cheap and reliable source of animals and large quantities of endothelial cells can be isolated for drug screening studies. On the other hand, the proteins expressed by porcine models differ in sequence from their mouse and human homologues [38] and this can result in affinity and transport rate differences, especially when the therapeutical antibody is designed to react with human or rodent homologues. Murine or human BEC models give preferable results in these types of study. The mouse bEnd.3 and human hCMEC/D3 cell lines have the advantage of originating from species which are thoroughly characterized and give data more comparable to the preclinical and clinical studies. For instance, the mouse bEnd.3 cell line provides useful reference information for the in vivo rodent models, while the human hCMEC/D3 cell line can predict the outcome for clinical studies [37]. Therefore, our comparative investigation has expanded previous knowledge on transcytosis capacity of these in vitro BBB models [13, 14, 28–32, 39] and provided a more stable platform to choose the most suitable model for investigations where the endo-lysosomal system is involved.

Our findings can also offer a basis to interpret differences in drug delivery properties of these models. Nonetheless, we should mention that the endo-lysosomal system of the BEC is a delicate fine-tuned network and components of the media and presence of astrocytes may influence this system [12]. In our previous study, we have e.g. investigated the effect of differentiation factors such as hydrocortisone, cAMP and the presence of astrocytes on primary porcine brain endothelial cells. We found that these factors changed the composition of the endo-lysosomal system. It would be interesting to analyse in future studies the influence of the other cells of the neurovascular unit in a complex 3D model, to give more reliable modelling of the in vivo situation. However, in the present study the selected models were used under conditions recommended for these cells by the suppliers and thereby used by most research groups. Also, we did not focus on interspecies differences of the in vitro models or compare primary models with cell lines from the same species, since those studies already exist in the literature. The pattern of transporters, receptors and tight junction proteins of the bEnd.3 mouse cell line has already been compared to that of primary mouse [40] and porcine BEC [13]. Similar comparison has been made of the human hCMEC/D3 cell line with primary human BEC [41] and with human BEC of stem cell origin [42]. However, these investigations did not extend to members of the endo-lysosomal system. Future studies need to elucidate the differences in the endo-lysosomal composition of primary BEC and cell lines originating from the same species.

## Conclusion

Taken together, our results will help increase understanding of the endo-lysosomal structure of BEC frequently used as in vitro models of the BBB. Thorough description of the vesicular transport system is highly important for better understanding of the intracellular mechanisms during receptor-mediated transcytosis. Data from our study may help to improve strategies to transverse the BBB more intelligently and to select the appropriate model for the experiment(s) of interest.

## Additional files

**Additional file 1.** List of antibodies applied for immunofluorescence and Western blot.

**Additional file 2.** Western blots showing the presence of different endosomal markers and β-actin in all the investigated groups.

**Additional file 3.** Representative confocal microscopy images of LysoSensor Green DND-189 (green)-loaded brain endothelial cells with or without bafilomycin. Nucleus is marked with blue. Magnification is 60×. Scale bar: 10 µm.

**Additional file 4.** Subcellular zones. Based on the lateral distance from the nucleus, subcellular zones were defined inside the cells. The juxtanuclear zone covers the area of nuclei and 1 µm around. The peripheral zone of the cells was delineated between 1 and 2 µm distance from the nucleus. The third zone covered mainly the projections (processes) of the cells, therefore it is mentioned as the zone of projection. Vesicles in the juxtanuclear zone are indicated with red, in the peripheral zone with green and in the processes with blue boxes. The nuclei are shown in white and interendothelial junctions are indicated with red. For better transparency the immunofluorescent staining of vesicles is not shown here.

## Data Availability

The datasets used and/or analysed during the current study are available from the corresponding author on reasonable request.

## Abbreviations

BBB: blood–brain barrier
BEC: brain endothelial cells
PBEC: primary porcine BECs
VPS: vacuolar protein sorting-associated protein
LAMP: lysosome-associated membrane protein
DMEM: Dulbecco’s Modified Eagle Medium
HEPES: (4-(2-hydroxyethyl)-1-piperazineethanesulfonic acid
PDS: plasma-derived bovine serum
FBS: fetal bovine serum
cAMP: 8-(4-chlorophenylthio)adenosine-3′,5′-cyclic monophosphate
PBS: phosphate buffered saline
RT: room temperature
^125^I-RAP: receptor associated protein radioactively labeled with Ci ^125^I
EEA1: early endosome antigen 1
TfR: transferrin receptor
RAB7: Ras-related protein 7
LRP-1: low-density lipoprotein receptor-related protein 1
