# Supplementary material for: The endo-lysosomal system of bEnd.3 and hCMEC/D3 brain endothelial cells
Source: Fluids Barriers CNS. 2019 May 30;16:14. doi: 10.1186/s12987-019-0134-9 (PMC6542060; doi:10.1186/s12987-019-0134-9)
Supplement: Supplementary file 1 — Additional file 1. List of antibodies applied for immunofluorescence and Western blot. [file 12987_2019_134_MOESM1_ESM.pdf]

Primary antibodies					
Marker for	Targeted protein	Abbreviation	Antibody	Manufacturer	Cat. no.
Early endosome	Early endosome antigen 1	EEA1	rabbit polyclonal anti-EEA1	Abcam	ab2900
Recycling endosome	Transferin receptor	TfR	rabbit polyclonal anti-TfR	Abcam	ab84036
Retromer positive vesicle	Vacuolar protein sorting-associated protein 35	VPS35	goat polyclonal anti-Vps35	Everest Biotech	EB06268
Late endosome	Ras-related protein 7	RAB7	rabbit polyclonal anti-Rab7	Abcam	ab137029
Lysosome	Lysosomal-associated membrane protein 1	LAMP1	rabbit polyclonal anti-Lamp1	Abcam	ab24170
Interendothelial junction	p-120 catenin	p120	mouse monoclonal anti-p120/catein	BD Bioscience	610133
	β-actin		mouse monoclonal anti-β-actin	Sigma	A5441

Secundary antibodies			
Applied for	Antibody	Manufacturer	Cat. no.
Immunofluorescent labelling of EEA1, TfR, RAB7, LAMP1	donkey anti-rabbit IgG Alexa Fluor 488 conjugate	Life Technologies	A21206
Immunofluorescent labelling of VPS35	donkey anti-goat IgG Alexa Fluor 488 conjugate	Invitrogen	A11055
Immunofluorescent labelling of p120	donkey anti-mouse IgG Alexa Fluor 568 conjugate	Invitrogen	A11031
Western blot for EEA1, TfR, RAB7, LAMP1	goat anti-rabbit-IgG HRP-linked	Cell Signalling	611620
Western blot for VPS35	rabbit anti-goat-IgG HRP-linked	Invitrogen	7074s
Western blot for β-actin	horse anti-mouse-IgG HRP-linked	Cell Signalling	7076s
