# Supplementary material for: The endo-lysosomal system of bEnd.3 and hCMEC/D3 brain endothelial cells
Source: Fluids Barriers CNS. 2019 May 30;16:14. doi: 10.1186/s12987-019-0134-9 (PMC6542060; doi:10.1186/s12987-019-0134-9)
Supplement: Supplementary file 2 — Additional file 2. Western blots showing the presence of different endosomal markers and β-actin in all the investigated groups. [file 12987_2019_134_MOESM2_ESM.pdf]

Marker for	Targeted protein	1 2 3	
Early endosomes	EEA1 (180 kDa)	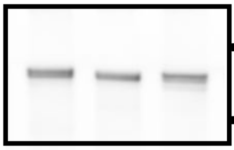	188 kDa<br>98 kDa
	β – actin (42 kDa)	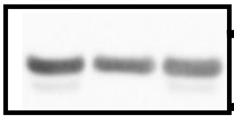	49 kDa<br>38 kDa
Recycling endosomes	TfR (62 kDa)	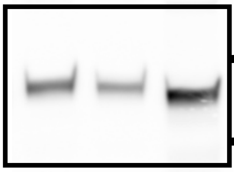	98 kDa<br>62 kDa
	β – actin (42 kDa)	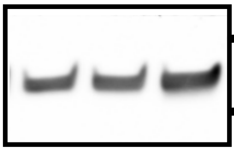	49 kDa<br>38 kDa
Late endosomes	RAB7 (23 kDa)	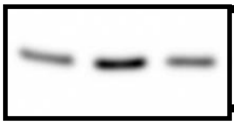	28 kDa<br>17 kDa
	β – actin (42 kDa)	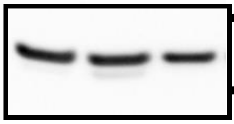	49 kDa<br>38 kDa
Lysosomes	LAMP1 (120 kDa)	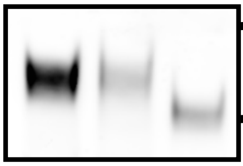	188 kDa<br>98 kDa
	β – actin (42 kDa)	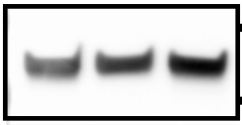	49 kDa<br>38 kDa
Retromer positive vesicles	VPS35 (85 kDa)	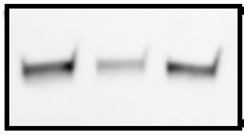	98 kDa<br>62 kDa
	β – actin (42 kDa)	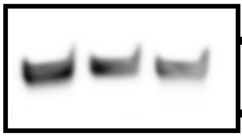	49 kDa<br>38 kDa

1; bEnd.3 2; PBEC 3; hCMBEC/D3
