# Supplementary figures and images for: The endo-lysosomal system of bEnd.3 and hCMEC/D3 brain endothelial cells
Source: Fluids Barriers CNS. 2019 May 30;16:14. doi: 10.1186/s12987-019-0134-9 (PMC6542060; doi:10.1186/s12987-019-0134-9)

**bEnd.3**

**hCMEC/D3**

**PBEC**

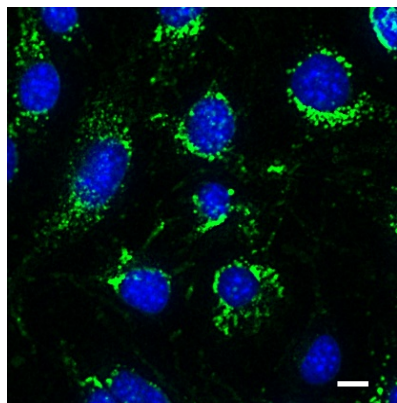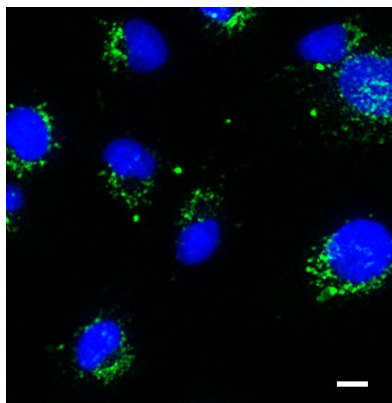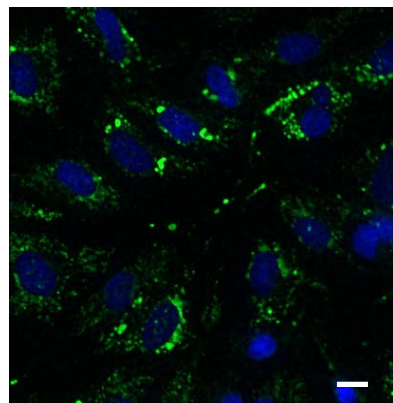

**Bafilomycin**

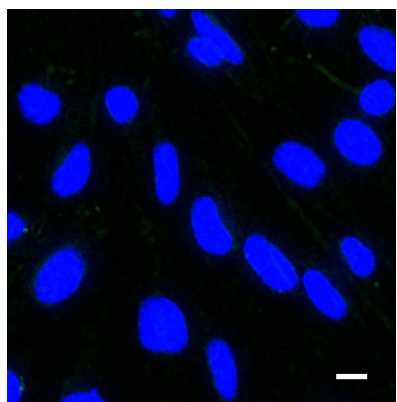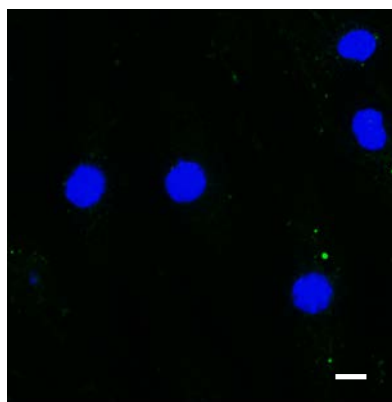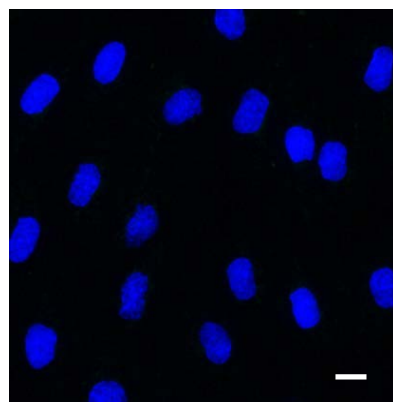

Supplement: Supplementary file 3 — Additional file 3. Representative confocal microscopy images of LysoSensor Green DND-189 (green)-loaded brain endothelial cells with or without bafilomycin. Nucleus is marked with blue. Magnification is 60×. Scale bar: 10 µm. [file 12987_2019_134_MOESM3_ESM.pdf]

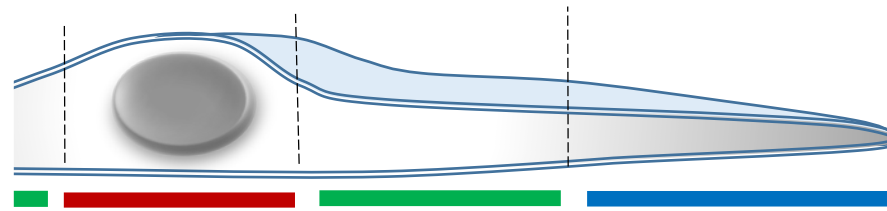

**b.End3**

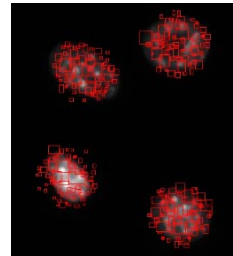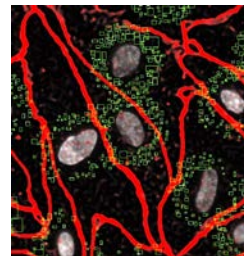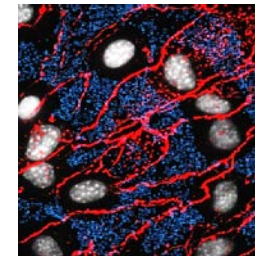

**hCMEC/D3**

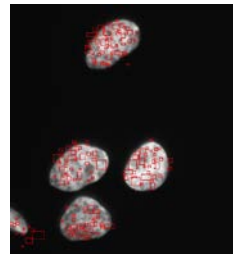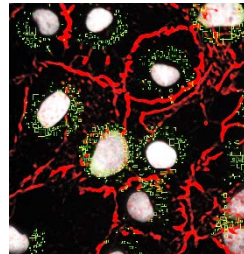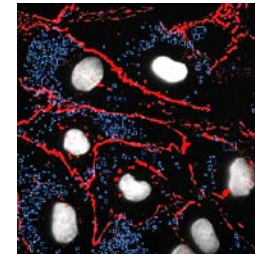

**PBEC**

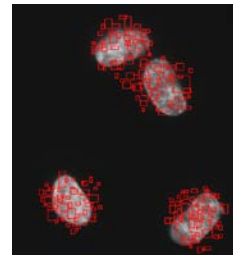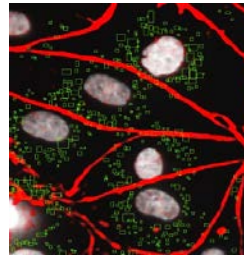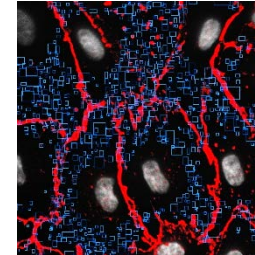

**Juxtanuclear  
zone**

**Peripheral  
zone**

**Projection  
zone**

Supplement: Supplementary file 4 — Additional file 4. Subcellular zones. Based on the lateral distance from the nucleus, subcellular zones were defined inside the cells. The juxtanuclear zone covers the area of nuclei and 1 µm around. The peripheral zone of the cells was delineated between 1 and 2 µm distance from the nucleus. The third zone covered mainly the projections (processes) of the cells, therefore it is mentioned as the zone of projection. Vesicles in the juxtanuclear zone are indicated with red, in the peripheral zone with green and in the processes with blue boxes. The nuclei are shown in white and interendothelial junctions are indicated with red. For better transparency the immunofluorescent staining of vesicles is not shown here. [file 12987_2019_134_MOESM4_ESM.pdf]
